# Recombinant Thrombomodulin Protects Mice against Histone-Induced Lethal Thromboembolism

**DOI:** 10.1371/journal.pone.0075961

**Published:** 2013-09-30

**Authors:** Mayumi Nakahara, Takashi Ito, Ko-ichi Kawahara, Mika Yamamoto, Tomoka Nagasato, Binita Shrestha, Shingo Yamada, Takahiro Miyauchi, Koji Higuchi, Toshihiro Takenaka, Tomotsugu Yasuda, Akira Matsunaga, Yasuyuki Kakihana, Teruto Hashiguchi, Yuichi Kanmura, Ikuro Maruyama

**Affiliations:** 1 Anesthesiology and Critical Care Medicine, Kagoshima University Graduate School of Medical and Dental Sciences, Kagoshima, Japan; 2 Systems Biology in Thromboregulation, Kagoshima University Graduate School of Medical and Dental Sciences, Kagoshima, Japan; 3 Cardiovascular, Respiratory, and Metabolic Medicine, Kagoshima University Graduate School of Medical and Dental Sciences, Kagoshima, Japan; 4 Cardiac Repair and Regeneration, Kagoshima University Graduate School of Medical and Dental Sciences, Kagoshima, Japan; 5 Emergency and Intensive Care Medicine, Kagoshima University Graduate School of Medical and Dental Sciences, Kagoshima, Japan; 6 Laboratory and Vascular Medicine, Kagoshima University Graduate School of Medical and Dental Sciences, Kagoshima, Japan; 7 Department of Biomedical Engineering, Osaka Institute of Technology, Osaka, Japan; 8 Shino-Test Corporation, Sagamihara, Japan; National Cerebral and Cardiovascular Center, Japan

## Abstract

**Introduction:**

Recent studies have shown that histones, the chief protein component of chromatin, are released into the extracellular space during sepsis, trauma, and ischemia-reperfusion injury, and act as major mediators of the death of an organism. This study was designed to elucidate the cellular and molecular basis of histone-induced lethality and to assess the protective effects of recombinant thrombomodulin (rTM). rTM has been approved for the treatment of disseminated intravascular coagulation (DIC) in Japan, and is currently undergoing a phase III clinical trial in the United States.

**Methods:**

Histone H3 levels in plasma of healthy volunteers and patients with sepsis and DIC were measured using enzyme-linked immunosorbent assay. Male C57BL/6 mice were injected intravenously with purified histones, and pathological examinations were performed. The protective effects of rTM against histone toxicity were analyzed both *in vitro* and in mice.

**Results:**

Histone H3 was not detectable in plasma of healthy volunteers, but significant levels were observed in patients with sepsis and DIC. These levels were higher in non-survivors than in survivors. Extracellular histones triggered platelet aggregation, leading to thrombotic occlusion of pulmonary capillaries and subsequent right-sided heart failure in mice. These mice displayed symptoms of DIC, including thrombocytopenia, prolonged prothrombin time, decreased fibrinogen, fibrin deposition in capillaries, and bleeding. Platelet depletion protected mice from histone-induced death in the first 30 minutes, suggesting that vessel occlusion by platelet-rich thrombi might be responsible for death during the early phase. Furthermore, rTM bound to extracellular histones, suppressed histone-induced platelet aggregation, thrombotic occlusion of pulmonary capillaries, and dilatation of the right ventricle, and rescued mice from lethal thromboembolism.

**Conclusions:**

Extracellular histones cause massive thromboembolism associated with consumptive coagulopathy, which is diagnostically indistinguishable from DIC. rTM binds to histones and neutralizes the prothrombotic action of histones. This may contribute to the effectiveness of rTM against DIC.

## Introduction

Sepsis is a leading cause of in-hospital death in developed countries [1,2]. This lethality has been thought to result from overwhelming inflammation during infection, and anti-inflammatory agents such as interleukin-1 receptor antagonists and neutralizing antibodies against tumor necrosis factor (TNF) have proven to be efficacious for the treatment of animal models of sepsis [3,4]. However, in humans most of these strategies have failed to improve the survival of patients with sepsis [5]. This is, in part, because levels of circulating cytokines such as TNF-α are much lower in patients with sepsis than in the animal models [1,5]. In the case of animal models, these were often exposed to large doses of lipopolysaccharide (LPS) or bacteria and died from a cytokine “storm”, which does not reflect the clinical picture in humans. This indicates that molecules other than classical pro-inflammatory cytokines may be responsible for the multiple organ failure and death in patients with sepsis.

Disseminated intravascular coagulation (DIC) is found in 25–50% of patients with sepsis and seems to be a strong predictor of mortality [6,7]. DIC is characterized by widespread microvascular thrombosis and profuse bleeding, as well as gross laboratory abnormalities including thrombocytopenia, prolonged clotting time, and elevated fibrin-related markers [6,8]. Intravascular thrombosis can interrupt the blood supply to organs, resulting in multiple organ failure. In addition, the reduction of platelets and coagulation factors may induce severe bleeding.

DIC is not a homogeneous disease but a syndrome, which is always secondary to an underlying disorder such as sepsis or trauma [6]. During infection, microbial pathogen-associated molecular patterns such as LPS activate pro-thrombotic responses as well as pro-inflammatory responses [9-11]. In addition, cellular injury can release endogenous damage-associated molecular patterns (DAMPs), including mitochondrial proteins and intra-nuclear high mobility group box 1 protein (HMGB1), which also activate pro-thrombotic and pro-inflammatory responses in the extracellular milieu [12-15]. Microvessel thrombosis may represent a physiological tool of host defense because it can suppress pathogen dissemination [16,17]. However, excessive pro-thrombotic reactions result in the development of DIC, and are therefore no longer beneficial to the hosts.

Recent studies have identified histones, the most abundant proteins in the nucleus, as a new class of DAMPs [18-21]. Extracellular histones promote neutrophil migration, platelet aggregation, and endothelial cell death [18,22,23]. Histones have been detected in the plasma of mice, baboons, and human patients with sepsis and trauma, and the total concentration of histones can reach 70, with that of histone H3 reaching 15 µg/ml [18,24]. Neutralizing antibodies to histones can rescue mice from lethal endotoxemia and peritonitis-associated sepsis, suggesting that extracellular histones are major mediators of death in sepsis [18,19]. However, the cellular and molecular basis of histone-induced lethality remains to be fully elucidated. Here we provide evidence that extracellular histones cause massive thromboembolism associated with consumptive coagulopathy. Furthermore, recombinant thrombomodulin (rTM), which has been approved for the treatment of DIC in Japan and is currently undergoing a phase III clinical trial in the United States, binds to histones and protects mice against histone-induced fatal thrombosis.

## Materials and Methods

### Measurement of plasma histone H3 levels

All experiments involving human blood conformed to the provisions of the Declaration of Helsinki and were approved by the Ethics Committee of Kagoshima University. Written informed consent for participation in this study was obtained from all individuals. Diagnoses of sepsis and DIC were made according to the guidelines of the Society of Critical Care Medicine Consensus Conference Committee and the diagnostic criteria established by the Japanese Association for Acute Medicine criteria (JAAM DIC criteria), respectively. Plasma samples were collected from 15 healthy volunteers and 26 patients with sepsis and DIC. Polystyrene microtiter plates were coated with anti-human histone H3 polyclonal antibody (Abcam, Cambridge, United Kingdom) and were incubated overnight at 2-8°C. The plates were washed three times, and the remaining binding sites were blocked by PBS containing 1% bovine serum albumin (BSA). The plates were washed again then incubated with the calibrator and samples for 24 hours at room temperature. The plates were washed again, and incubated with anti-human histone H3 peroxidase-conjugated polyclonal antibody (Abcam) for 2 hours at room temperature. After another washing step, the chromogenic substrate 3,3’,5,5’-tetra-methylbenzidine (Dojindo Laboratories, Kumamoto, Japan) was added to each well. The reaction was terminated by the addition of 0.35 mol/L Na _2_SO_4_, and the absorbance at 450 nm was read using a microplate luminescence reader (Bio-Rad, Hercules, CA).

### Mice

Experiments involving animals were approved by the Institutional Animal Care and Use Committee of Kagoshima University, Kagoshima, Japan. Male C57BL/6 mice (9 to 13 weeks old, Kyudo, Fukuoka, Japan) received a single tail vein injection of purified unfractionated calf thymus histones (20-95 µg/g, Sigma-Aldrich, St Louis, MO), which contained little or no endotoxin (1.25 ± 0.2 pg/mg histones). The purity of the histones was further evaluated by sodium dodecyl sulfate polyacrylamide gel electrophoresis followed by Coomassie brilliant blue staining (Figure S1A in File S1), and the identity of each band was confirmed by western blotting. Survival was monitored for up to 96 hours. Blood and lung samples were collected from volatile-anesthetized mice 10 minutes after histone injection. In some experiments, recombinant human soluble thrombomodulin (rTM; Asahi Kasei Pharma Corporation, Tokyo, Japan) was administered as a bolus injection into the tail vein 30 minutes before histone injection. The amino acid composition of soluble thrombomodulin has been described previously [25]. Platelets were depleted by intravenous application of anti-GP Ib antibodies (2 µg/g, Emfret Analytics, Eibelstadt, Germany) 2 hours before the histone injection (80 µg/g).

### Electrocardiographic and echocardiographic analyses

Mice were anesthetized via an intraperitoneal injection of ketamine (100 µg/g) and xylazine (10 µg/g). Electrocardiogram (ECG) measurement was performed using a Powerlab/8S multi-channel digital data recorder (A D Instruments, Sydney, Australia), and the voltage between the right limb and the feet (lead II) was recorded. Transthoracic echocardiography was performed using an Acuson Sequoia Ultrasound System (Siemens Medical Systems, Mountain View, CA) in mice anesthetized with an intraperitoneal injection of pentobarbital (50 µg/g). Right ventricular diastolic dimension was measured in the standard parasternal long axis view using conventional M-mode echocardiography.

### Blood tests

Blood samples were collected from volatile-anesthetized mice 10 minutes after histone injection, and coagulation was prevented by addition of one-tenth volume of either 3.13% sodium citrate or EDTA. Prothrombin time (PT) and activated partial thromboplastin time (APTT) were measured by standard methods using a KC1 Delta automatic coagulometer with an electromechanical clot detection instrument (Trinity Biotech, Bray, Ireland). Complete blood counts were performed with EDTA-anti-coagulated blood, using an automated counting device XE-5000 (Sysmex Corporation, Kobe, Japan). Basic biochemical examinations were performed using a LABOSPECT 008 biochemical analyzer (Hitachi, Tokyo, Japan).

### Immunofluorescent analyses

For *in vivo* labeling of platelets, mice were injected intravenously with DyLight 488-conjugated monoclonal antibodies against GP Ibβ (0.15 µg/g, Emfret Analytics) 30 minutes before histone injection. Lung samples were collected 10 minutes after histone injection (75 µg/g), fixed in 4% paraformaldehyde, equilibrated in 10-30% sucrose, and frozen in optimal cutting temperature compound (Sakura Finetech, Tokyo, Japan). Frozen sections (4 µm) were probed with anti-fibrinogen/fibrin IgG (Dako Cytomation, Glostrup, Denmark) followed by Alexa-Fluor 594-conjugated goat anti-rabbit IgG (Invitrogen, Carlsbad, CA). Nuclei were stained with 4',6-diamidino-2-phenylindole (DAPI), and the sections were analyzed using an LSM700 confocal laser microscope (Zeiss, Oberkochen, Germany).

### Immunohistochemical analyses

Intravascular von Willebrand factor (VWF)-rich thrombi were stained according to a previously described method [26]. Briefly, lung specimens were probed with rabbit polyclonal anti-VWF antibody (CHEMICON International Inc., Temecula, CA) followed by Histofine simple stain mouse MAX-PO (R) (Nichirei, Tokyo, Japan). Peroxidase activity was visualized using diaminobenzidine (DakoCytomation, Carpinteria, CA), and the slides were lightly counterstained with Lillie-Meyer’s hematoxylin (Wako, Osaka, Japan). Photographs were taken using a Zeiss Axiophot microscope with an AxioCam MRc5 camera equipped with AxioVision Release 4.6 software (Carl Zeiss, Oberkochen, Germany).

### *In vitro* platelet aggregation assays

Blood samples were taken from four healthy volunteers, who had not taken any medications that might affect platelet function or coagulation in the preceding 2 weeks. Washed platelets resuspended in Tyrode-HEPES buffer (pH 7.35) or platelet-rich plasma anticoagulated with either sodium citrate or hirudin were stimulated with recombinant human histone H3 or H4 (New England Biolabs, Ipswich, MA), or with collagen. In some experiments, rTM was pre-incubated with histone H3 or H4, or with collagen, for 30 minutes. Platelet aggregation was optically monitored by a light transmission aggregometer (MCM Hema Tracer 313M; SSR Engineering, Tokyo, Japan).

### Binding assays with a quartz crystal microbalance (QCM) twin sensor system

The interaction between histones and rTM was assessed using a NAPiCOS Auto QCM twin sensor system (Nihon Dempa Kogyo Co., Tokyo, Japan) according to a previously described method [27]. Briefly, one channel of a sensor chip was coated with histones (1 mg/ml). The second channel on the same sensor chip was coated with BSA (1 mg/ml, Sigma-Aldrich, St Louis, MO) and served as a reference. The sensor chip was washed five times with phosphate-buffered saline (PBS; pH 7.4), placed into the NAPiCOS Auto instrument, perfused with PBS until a stable baseline was obtained, and then perfused with rTM (1 mg/ml). The interaction between molecules was recognized as the change in frequency of a quartz crystal resonator. All experiments were carried out at 25°C with a flow rate of 5 µl/min.

### Immunoblotting

Serum histone H3 levels were determined by immunoblotting analysis using anti-histone H3 antibody (Santa Cruz Biotechnology, Santa Cruz, CA) with reference to standard curves of recombinant histone H3 (0-10 µg/ml) diluted in normal mouse serum.

### Statistical analyses

Data are presented as mean ± SD. Statistical analyses were performed using Dunnett’s test unless otherwise stated. Survival rate was analyzed by the Kaplan-Meier method. A probability of < 0.05 was considered significant.

## Results

### Histone H3 levels in plasma of patients with sepsis and DIC

**Figure 1:**
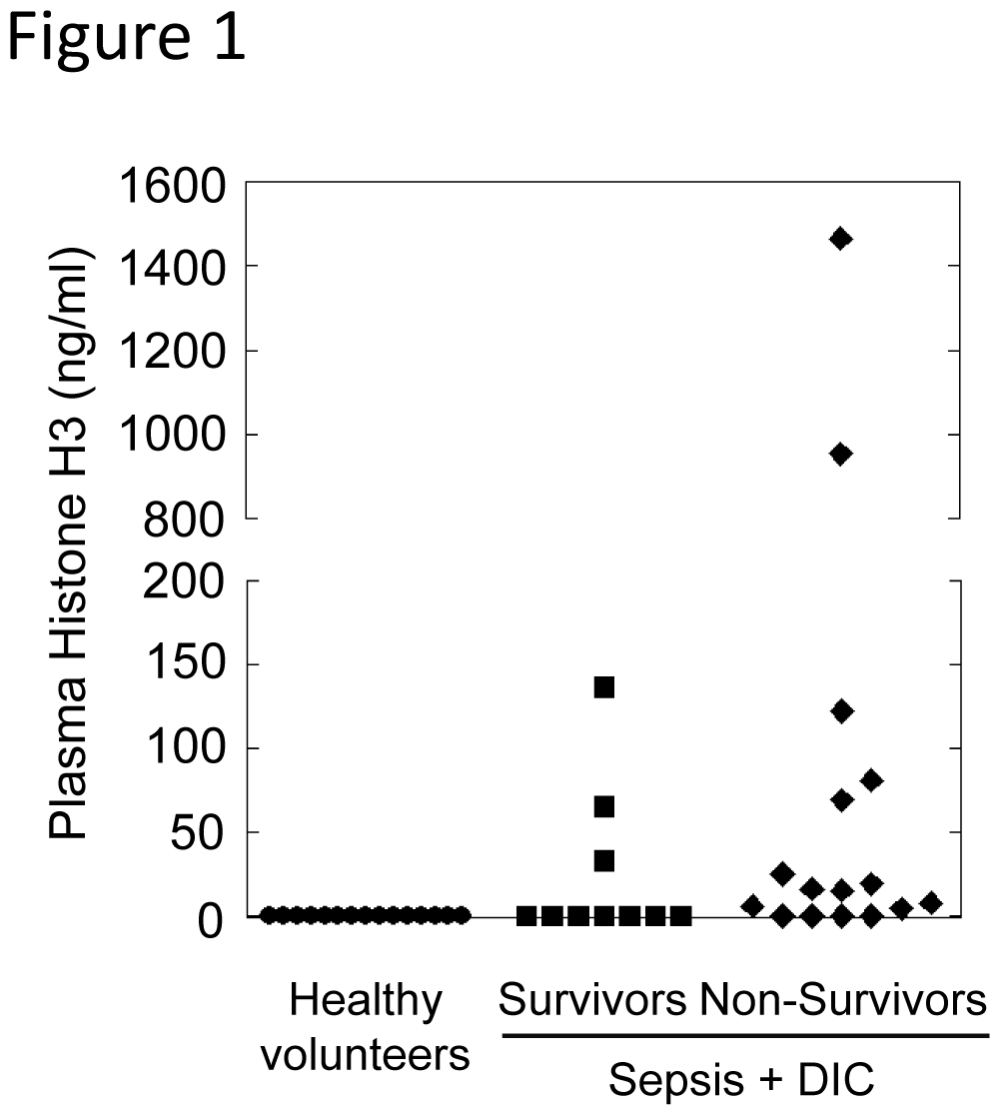
Histone H3 levels, as measured by ELISA, in plasma of patients with sepsis and DIC. Plasma histone H3 levels were significantly higher in non-survivors (n = 16, minimum = 0, maximum = 1464.4, median = 15.5) compared with healthy volunteers (n = 15, minimum = 0, maximum = 0) as analyzed by Steel-Dwass test.

Recent studies have shown that extracellular histones play an important role in the pathogenesis of sepsis, ischemia-reperfusion injury, and deep vein thrombosis in mice [18,20,28,29]. To examine the possible participation of extracellular histones in the pathogenesis of human sepsis, we studied 15 healthy volunteers and 26 patients with sepsis and DIC. As shown in Figure 1, histone H3 was not detectable in plasma of healthy volunteers, but significant levels were observed in patients with sepsis and DIC, especially in non-survivors. These data support the previous finding that extracellular histones are mediators of death in sepsis [18].

### Extracellular histones trigger platelet aggregation, leading to fatal thromboembolism in mice

**Figure 2:**
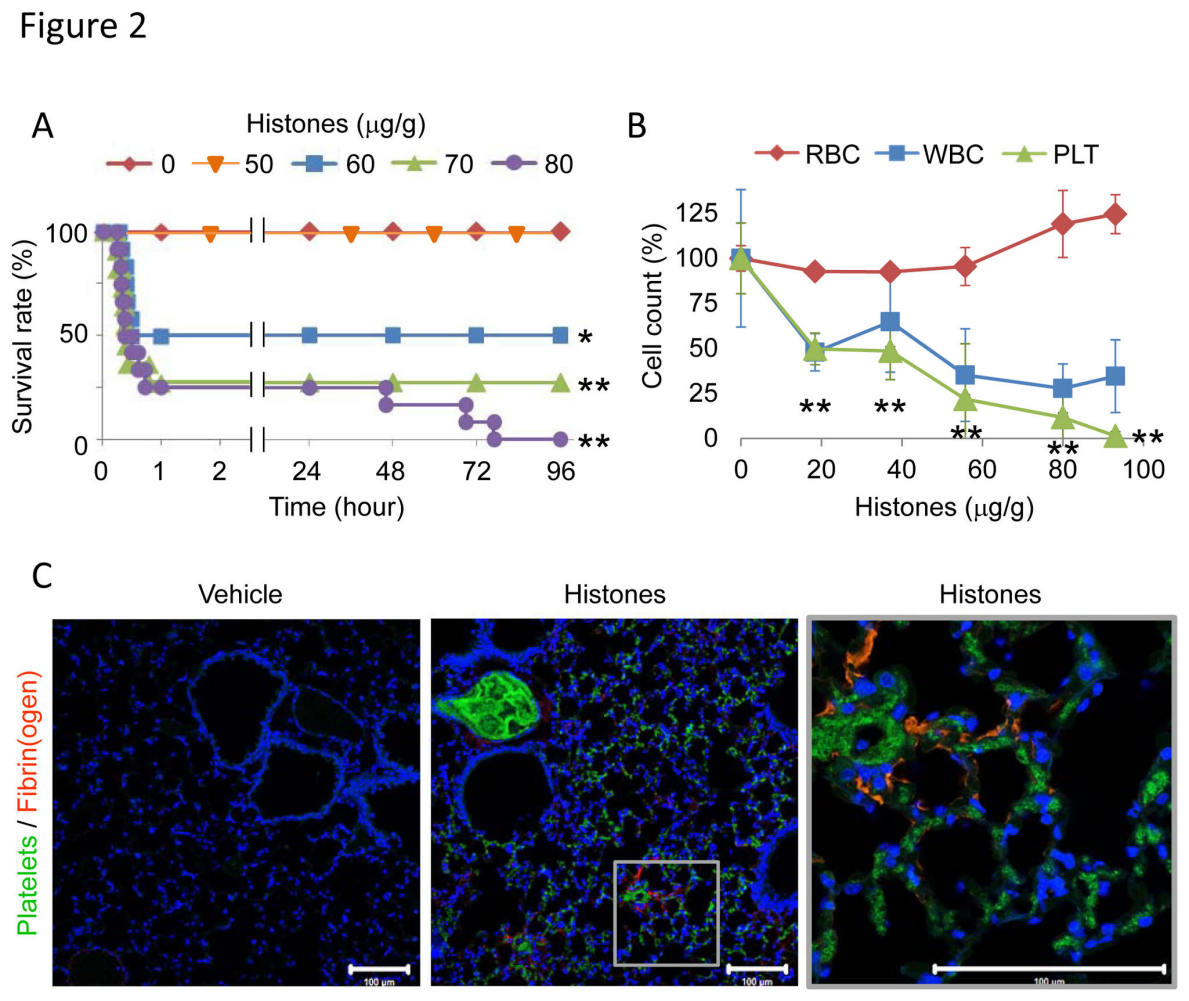
Extracellular histones cause fatal thromboembolism in mice. (A) The lethal effect of extracellular histones. Mice were intravenously injected with histones (0-80 µg/g, n = 7-12 per group), and survival was analyzed. (B) Histone-induced thrombocytopenia. Numbers of platelets (PLT), red blood cells (RBC), and white blood cells (WBC) in blood 10 min after infusion with histones (0-95 µg/g, n = 3-7 per group, mean ± S.D.) are shown. Data are presented as percentage of the vehicle group (0 µg/g histones). (C) Distribution of DyLight488-labeled platelets and Alexa-Fluor 594-labeled fibrin(ogen) in lung tissue 10 min after infusion with vehicle or 75 µg/g histones. Nuclei were stained with DAPI. Representative images of n = 4. Scale bar = 100 µm. * *P* < 0.05 and ** *P* < 0.01 compared with the vehicle group.

To explore how extracellular histones impact disease conditions, we injected mice intravenously with purified histones. Injection of 20-80 µg/g of histones resulted in serum histone H3 levels of 1-10 µg/ml (Figure S1B in File S1), which is comparable to those seen in mice and human patients with sepsis (Figure 1) [18,24]. Half of the mice died after being injected with 60 µg/g of histones, and all mice died after injection with 80 µg/g of histones (Figure 2A). Histones caused thrombocytopenia, but not erythropenia, in a dose-dependent manner, and completely depleted circulating platelets when larger doses were injected (Figure 2B). Neither platelets nor platelet aggregates were observed in blood smears from histone-injected mice (Figure S2 in File S1). Instead, fragmented red blood cells were observed, suggesting that histone-induced thrombosis may disturb the microcirculation [6]. To investigate the effect of histones on platelets, we labeled platelets *in vivo* with DyLight488-conjugated immunoglobulin against murine platelet GP Ibβ, which does not interfere with platelet adhesion and aggregation. In response to histones, DyLight488-labeled platelets aggregated and diffusely obstructed pulmonary microvessels (Figure 2C). In addition, fibrin was deposited around platelets (Figure 2C), consistent with previous reports showing that extracellular histones activate coagulation via a platelet polyphosphate-dependent mechanism [30]. Histone H3 and H4 were sufficient to induce platelet aggregation *in vitro* (Figure S3A in File S1), suggesting that platelet aggregation might be associated with histone-induced thrombocytopenia and thromboembolism. Histone-induced platelet aggregation was attenuated in the presence of citrate (Figure S3B in File S1). This may suggest either that calcium ions are important in histone-induced platelet aggregation or that citrate directly inhibits histone activity.

**Figure 3:**
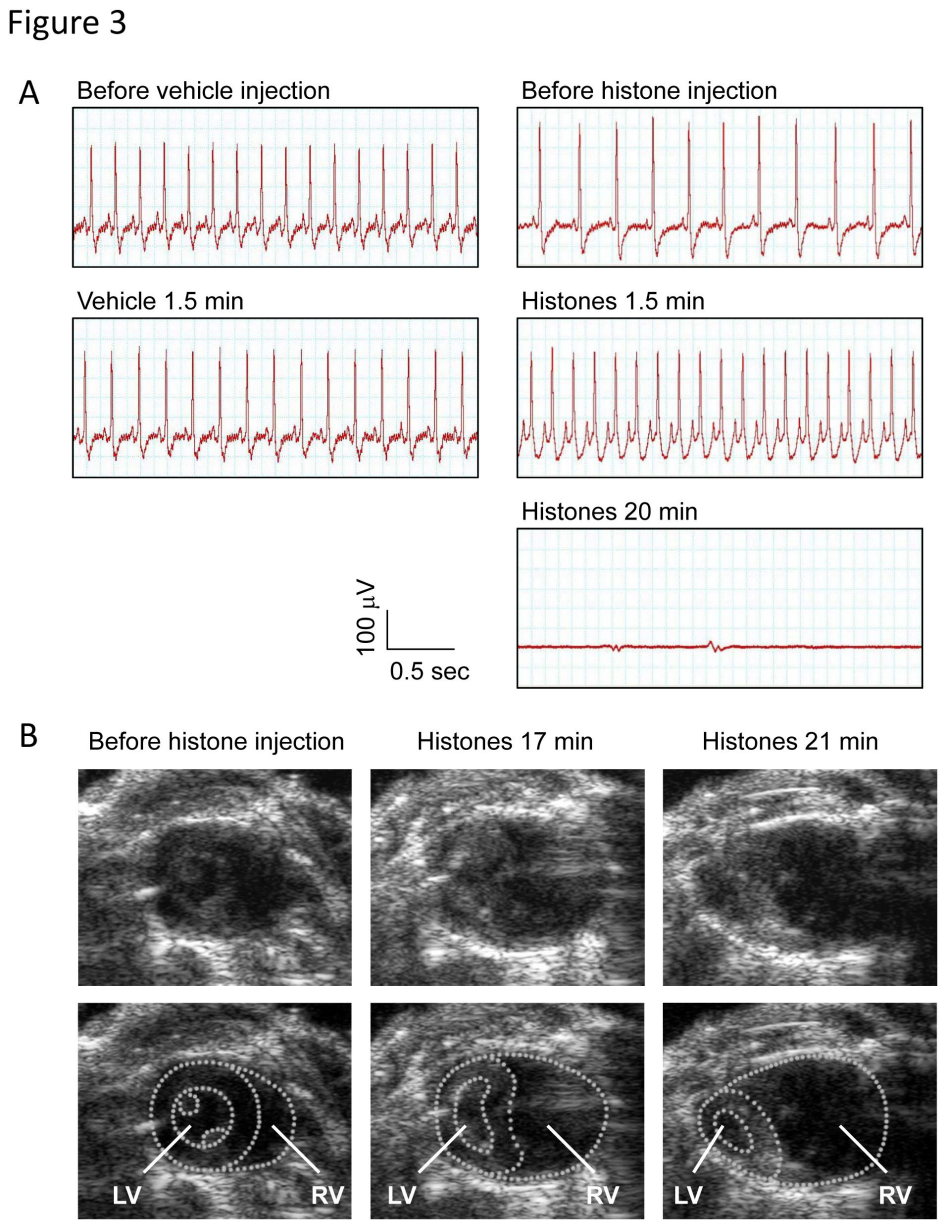
Extracellular histones cause acute right-sided heart failure and ventricular arrest. (A) ECG in mice intravenously injected with vehicle or histones (80 µg/g). The voltage between the right limb and the feet (lead II) was recorded. Data presented are representative of three independent experiments. (B) Still images from Movies S1-S3. Transthoracic echocardiography was performed in mice before and after intravenous injection of histones. Extracellular histones caused dilatation of the right ventricle (RV) and displacement of the interventricular septum toward the left ventricle (LV).

Next, we performed electrocardiographic and echocardiographic monitoring to evaluate the potentially fatal role of histone-induced thromboembolism. Histone injection resulted in a tall, peaked P-wave (Figure 3A), dilatation of the right ventricle (Figure 3B), and displacement of the interventricular septum towards the left ventricle (Movies S1 and S2), suggesting that histone-induced thromboembolism is not a minor event but a severe disorder that results in acute pulmonary hypertension and right-sided heart failure. Furthermore, the right-sided heart failure was followed by ventricular arrest (Figure 3A and Movie S3) within 30 min, which is consistent with the time course of histone-induced death (Figure 2A). These findings indicate that pulmonary thromboembolism was closely associated with histone-induced death.

To further examine the causal relationship between platelet-rich thrombi and histone-induced death, we removed platelets from the circulation before injecting histones. As described in previous reports [31,32], anti-GPIbα antibodies reduced the number of platelets in the circulating blood by almost 95%. These platelet-depleted mice were protected from death in the first 30 min after histone challenge (Figure S4 in File S1). However, the protective effect of platelet depletion disappeared over time, indicating that extracellular histones mediate death via several distinct mechanisms, where platelet-mediated microvessel occlusion plays an important role in the early phase death and other factors may be responsible for the later phase death. Taken together, our data indicate that extracellular histones trigger platelet-rich thrombus formation in pulmonary microvessels, resulting in acute right-sided heart failure and ventricular arrest, and this mechanism is responsible for histone-induced death in the early phase in mice.

### Extracellular histones cause consumptive coagulopathy in mice

**Figure 4:**
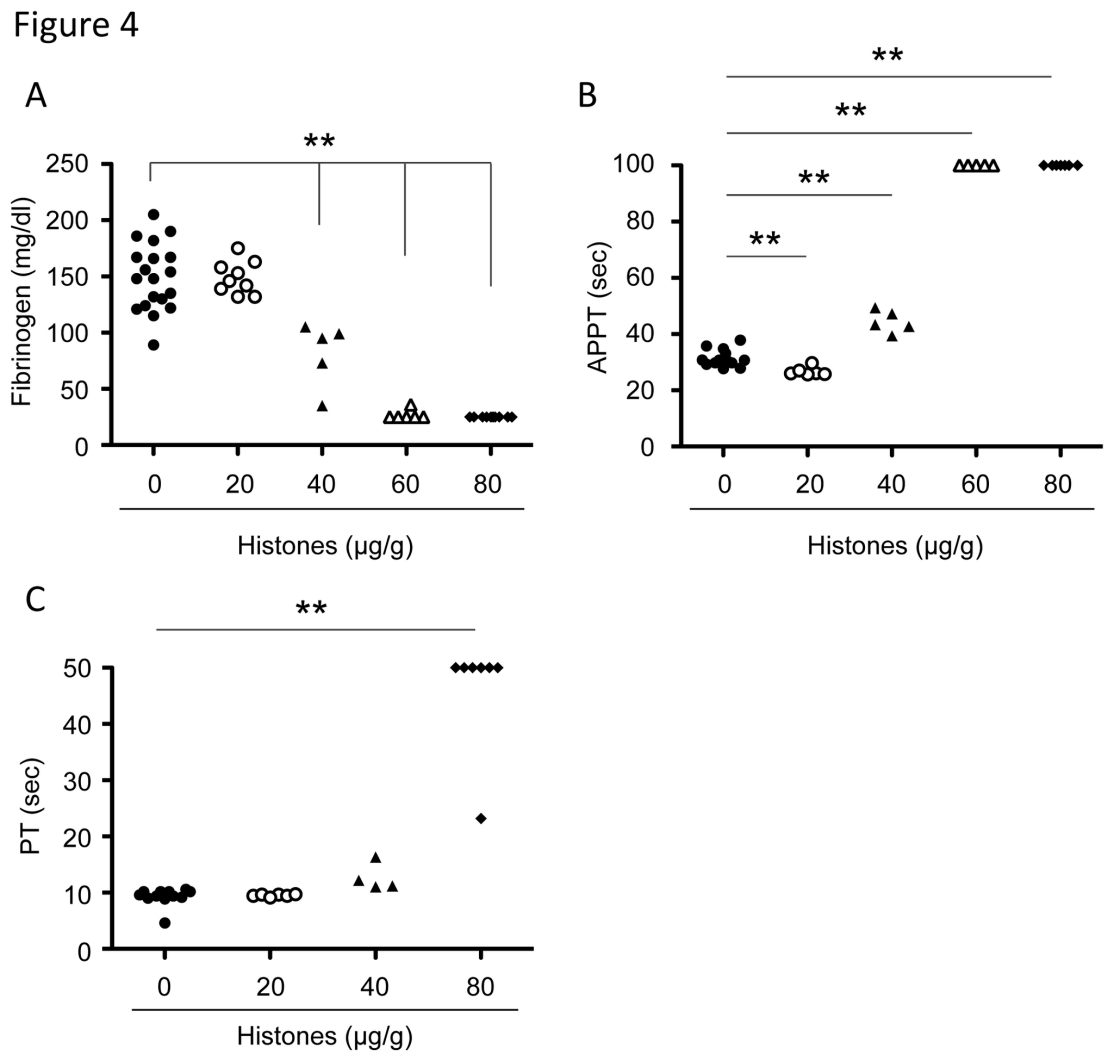
Extracellular histones cause consumptive coagulopathy. Plasma fibrinogen (A), APTT (B), and PT (C) of mice 10 minutes after injection with vehicle or histones (20-80 µg/g, n = 4-10). ** *P* < 0.01 compared with the vehicle group.

We examined the effects of extracellular histones on coagulation pathways. In line with previous reports [18,30], histone injection resulted in fibrin deposition around platelet-rich thrombi (Figure 2C). Fibrinogen levels were decreased when mice were injected with histones at a dose of 40 µg/g or higher (Figure 4A), while APTT and PT were prolonged (Figure 4B-C). Interestingly, APTT was reduced when mice were injected with 20 µg/g histones (Figure 4B). These results suggest that coagulation factors might be primed in the presence of a relatively small amount of histones, but might be fully activated and severely depleted in the presence of an excess of histones.

### Recombinant thrombomodulin (rTM) protects mice against histone-induced fatal thromboembolism

**Figure 5:**
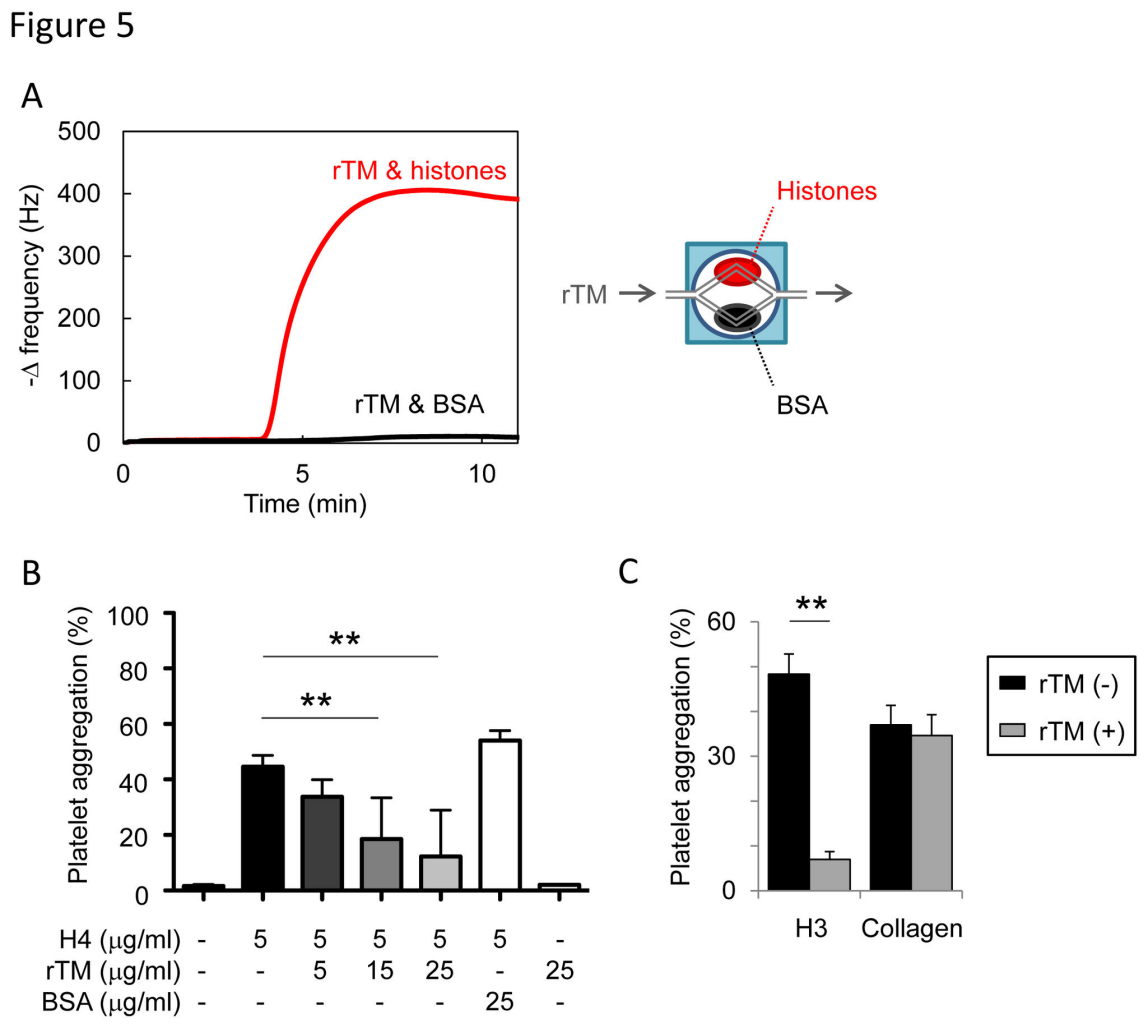
Recombinant thrombomodulin (rTM) suppresses the activity of extracellular histones. (A) Binding assays with a quartz crystal microbalance (QCM) twin sensor system. Two channels of a sensor chip were coated with either histones (1 mg/ml) or BSA (1 mg/ml). The sensor chip was placed into the NAPiCOS Auto and then perfused with rTM (1 mg/ml). The interaction between molecules was recognized as the change in frequency of a quartz crystal resonator. (B) Inhibition of histone H4-mediated platelet aggregation by rTM. Washed platelets were stimulated with histone H4 (5 µg/ml) preincubated with rTM (5-25 µg/ml) or BSA (25 µg/ml). rTM, but not BSA, inhibited histone-induced platelet aggregation (n = 3-7 per group, mean ± S.D.). (C) Inhibition of histone H3-induced platelet aggregation by rTM. Washed platelets were stimulated with histone H3 (25 µg/ml) or collagen (1.44 µg/ml) in the presence or absence of rTM (15 µg/ml). rTM inhibited histone-induced platelet aggregation, but not collagen-induced platelet aggregation. Representative data of three independent experiments are shown. ** *P* < 0.01 compared with the histone-alone group.

**Figure 6:**
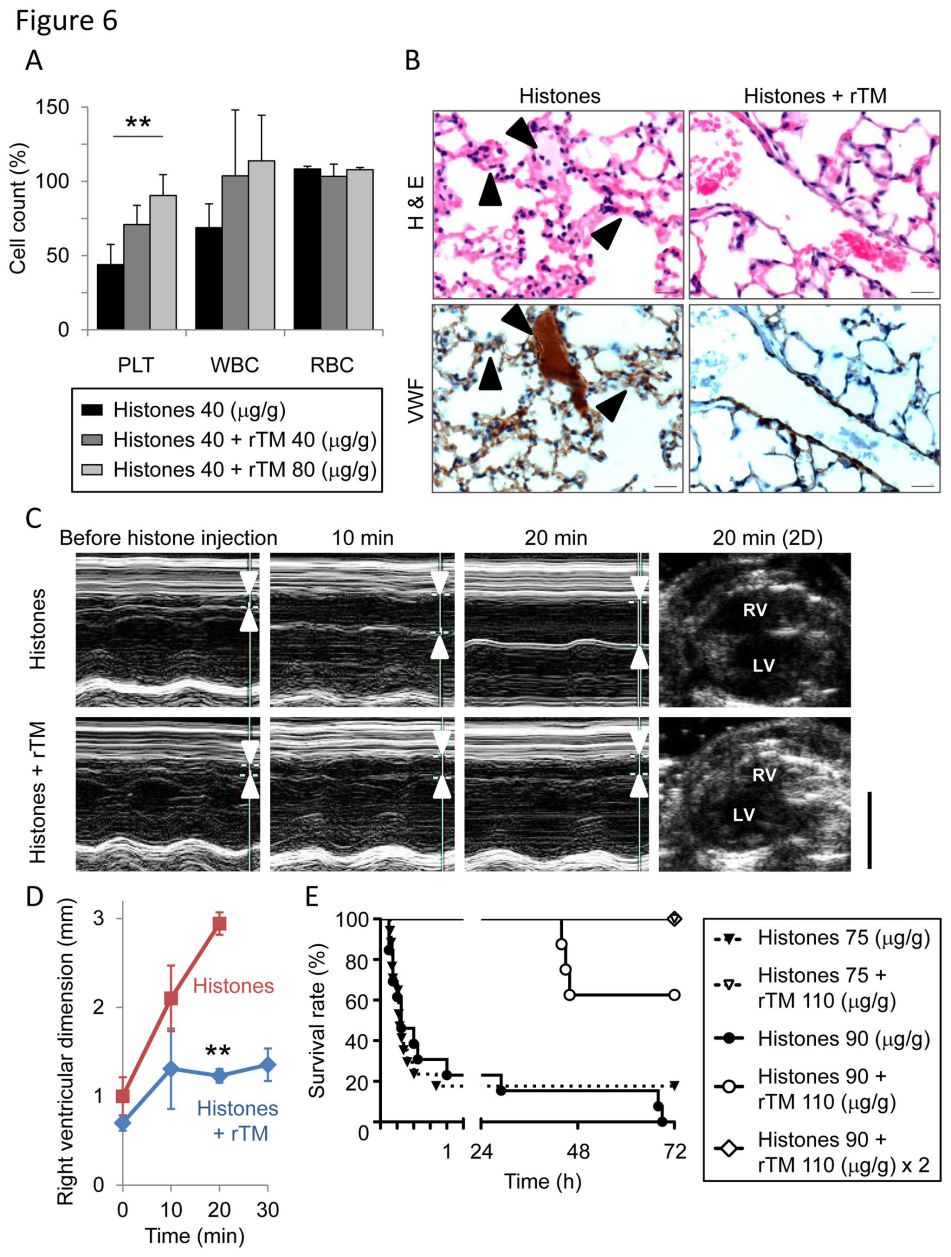
Recombinant thrombomodulin (rTM) protects mice against histone-induced fatal thrombosis. (A) The protective effect of rTM on histone-induced thrombocytopenia. Pretreatment with rTM (40 or 80 µg/g) 30 min before histone injection (40 µg/g) prevented histone-induced thrombocytopenia in mice (n = 3-4 per group, mean ± S.D.). ** *P* < 0.01 compared with the histone-alone group. (B) The protective effect of rTM on histone-induced VWF-rich thrombus formation. Arrowheads indicate intravascular thrombi. Pretreatment with rTM (110 µg/g) 30 min before histone injection (75 µg/g) suppressed histone-induced VWF-rich thrombus formation. Representative images of n = 5 (histone-alone) and n = 4 (histones + rTM). Scale bar = 20 µm. (C) The protective effect of rTM on histone-induced right-sided heart failure. Right ventricular diastolic dimension was measured before and 10, 20, and 30 minutes after histone injection using conventional M-mode echocardiography. Arrowheads indicate right ventricles. Pretreatment with rTM suppressed histone-induced dilatation of the right ventricle. RV: right ventricle. LV: left ventricle. Representative images of n = 4 are shown. Scale bar = 5 mm. (D) Quantitation of (C). Pretreatment with rTM (110 µg/g) 30 minutes before histone injection (75 µg/g) suppressed histone-induced dilatation of the right ventricle. The data of histone-alone group in 30 minutes are lacking because none of the mice in this group survived for up to 30 minutes after histone injection. ** *P* < 0.01 compared with the histone-alone group. (E) The protective effect of rTM on histone-induced lethality. Mice were treated with a single injection of 110 µg/g of rTM 30 minutes before histone injection (75 or 90 µg/g) or double injection of 110 µg/g of rTM 30 minutes before and 90 minutes after histone injection (90 µg/g). rTM therapy significantly improved survival of histone-injected mice (n = 8-21 per group, *P* < 0.01 compared with mice injected with histone alone).

Histone-injected mice displayed signs of DIC, including thrombocytopenia (Figure 2B), prolonged PT (Figure 4C), decreased fibrinogen (Figure 4A), fibrin deposition in capillaries (Figure 2C), and bleeding (Figure S5A in File S1), which meet the diagnostic criteria for overt DIC [33]. rTM, a newly approved drug for DIC in Japan [34,35], is known to exert anti-DIC effects through promoting protein C activation; however, little is known about its effect on extracellular histones. As suggested by a previous report [36], rTM bound to histones (Figure 5A). rTM inhibited histone H3- and H4-mediated platelet aggregation, but not collagen-mediated platelet aggregation (Figure 5B-C), indicating that rTM inhibits the activity of histones, rather than platelet aggregation itself. *In vivo*, a single injection of rTM protected mice against histone-induced thrombocytopenia (Figure 6A), VWF-rich intravascular thrombus formation (Figure 6B), dilatation of the right ventricle (Figure 6C-D), pulmonary hemorrhage (Figure S5A in File S1), and death (Figure 6E). Thus, rTM can suppress the activity of extracellular histones both *in vitro* and *in vivo*, and this may contribute to the effectiveness of rTM against thrombotic disorders.

## Discussion

Recent studies have shown that histones can be released into the extracellular space and act as major mediators of death in sepsis [18,24]. In this study we have shown that significant levels of extracellular histone H3 are present in plasma of human patients with sepsis and DIC. Furthermore, extracellular histones trigger platelet aggregation, resulting in thrombotic occlusion of microvessels and consumptive coagulopathy resembling DIC in mice. The consumptive coagulopathy seen in our murine model differs from traditional DIC in terms of time course (minutes *vs.* days) and extent of disorder (massive thrombosis in lung *vs.* systemic thrombosis) although these two conditions are difficult to distinguish by clinical laboratory findings. A similar example is that patients with massive pulmonary embolism develop a state of consumptive coagulopathy resembling DIC [37]. In these cases, extended intravascular coagulation may be a better term than DIC [38].

It is not yet clear where extracellular histones come from during sepsis. There are two proposed sources: one is necrotic cells and the other is neutrophils [19]. In response to endotoxin, platelets bind to neutrophils, promoting the release of neutrophil extracellular traps (NETs), which then ensnare bacteria in septic blood [39]. Histones are important constituents of NETs, and exert antibacterial and prothrombotic effects [16,22,40]. In plasma from baboons challenged with *E. coli*, the total concentration of histones reached 70 µg/ml, and that of histone H3 reached 15 µg/ml [18]. These concentrations are comparable to those required to induce platelet aggregation in our *in vitro* experiments (Figure S3 in File S1) and thrombosis in our in vivo experiments (Figure 2). Serum histone H3 levels in mice 10 minutes after a 40 µg/g histone injection were 3-6 µg/ml (Figure S1B in File S1) although the theoretical maximum concentration of serum histone H3 is 240 µg/ml. This discrepancy may be due to sequestration of histones by platelet-rich thrombi and rapid clearance of histones from circulation during this 10-minute period. Sequestration and rapid clearance could also occur in patients with sepsis/DIC, and thus it is suggested that concentrations of histone H3 could be much higher around sites of injuries than those in serum or plasma. It will also be important to clarify the prognostic value of other members of histone family, such as histone H4, in sepsis. So far, we have not been able to measure serum or plasma concentrations of histone H4 because antibodies against histone H4 are not sufficiently sensitive to detect plasma or serum histone H4.

Histone-induced death follows a biphasic pattern, with either rapid progression to death within an hour, or a later development of death over days (Figure 2A). Extracellular histones trigger platelet aggregation, leading to thrombotic occlusion of pulmonary capillaries and right-sided heart failure within 30 min. Some mice suffered sudden cardiac arrest before developing right-sided heart failure (data not shown) indicating that direct cardiotoxicity or hyperkalemia may be associated with a proportion of histone-induced early phase death. However, in most cases platelet-mediated thromboembolism is responsible for the early phase death because dilatation of the right ventricle and displacement of the interventricular septum toward the left ventricle preceded ventricular arrest. This notion is further supported by the finding that platelet depletion protected mice from histone-induced death in the first 30 min. In contrast, late phase death is caused by factors other than platelets. Recent studies have shown that extracellular histones are toxic toward cells, including endothelial cells and hepatocytes [18,20]. Consistent with this, we found that extracellular histones induced tissue damage, as evidenced by elevation of transaminase, lactate dehydrogenase, and creatine phosphokinase (data not shown). Multiple organ failure caused by direct histone cytotoxicity may be responsible for the later phase death.

rTM was recently approved for the treatment of DIC in Japan [34,35]. Structurally, rTM is composed of an N-terminal lectin-like domain, followed by six epidermal growth factor (EGF)-like domains, and an *O*-glycosylation-rich domain. The N-terminal lectin-like domain is important to inactivate pro-inflammatory HMGB1 [34,41] whereas the fourth, fifth, and sixth EGF-like domains of rTM are critical to form a high-affinity complex with thrombin and to activate anticoagulant protein C [34,41]. Activated protein C (APC) exerts anticoagulant effects through inactivating coagulation factors Va and VIIIa, and cytoprotective effects in part through degradation of extracellular histones [18,19]. In this study we found that rTM suppressed histone-mediated platelet aggregation and fatal thromboembolism. This suppressive effect of rTM against histones may be mediated in part through APC; however, rTM may also have a direct effect because it bound to histones (Figure 5A) and suppressed histone-mediated platelet aggregation in the absence of APC (Figure 5B-C). Furthermore, histone H3 degradation products were not detected in histone-injected mice treated with rTM (Figure S5B in File S1), indicating that APC-mediated histone degradation is not responsible for the protective effects of rTM in our mouse model. The precise domain of rTM required for the interaction with histones remains an open question. The fourth, fifth, and sixth EGF-like domains are candidates because these domains contain negatively charged domains [42] and may interact with positively charged histones. Another candidate is the N-terminal lectin-like domain where a DNA-binding protein HMGB1 interacts [43]. Further investigations are needed to clarify the structural basis for binding of rTM and histones.

## Conclusions

Extracellular histones are not detectable in plasma of healthy volunteers; however, significant levels are present in plasma of patients with sepsis and DIC. Extracellular histones cause massive thromboembolism associated with consumptive coagulopathy, which is diagnostically indistinguishable from DIC. rTM binds to histones and protects mice against histone-induced fatal thrombosis.

## Supporting Information

File S1 **Supporting Figures.** **Figure S1.** (A) The purity of unfractionated calf thymus histones used in this study. Purified unfractionated calf thymus histones (Hs), recombinant histone H3, H4, H2A, and H2B were subjected to SDS-PAGE followed by CBB staining (left panel). M: Molecular weight markers. The identity of each band was confirmed by immunoblotting (right panel). (B) Serum histone H3 levels in mice 10 minutes after histone injection (0-80 µg/g body weight). Serum histone H3 levels were determined by immunoblotting analysis with reference to standard curves of recombinant histone H3 diluted in normal mouse serum at the indicated concentrations. Injection of 20-80 µg/g of histones resulted in serum histone H3 levels of 1-10 µg/ml. **Figure S2.** Blood smears of mice 10 minutes after histone injection. Histone injection (80 µg/g) eliminated platelets and yielded fragmented red blood cells (arrow heads). Scale bars = 20 µm. **Figure S3.** Histone-induced platelet aggregation. (A) Washed platelets were stimulated with histone H4 or H3 (10-30 µg/ml). Representative data of three experiments are shown. (B) Platelet-rich plasma anticoagulated with citrate (left panel) or hirudin (right panel) was stimulated with histone H4 (30-100 µg/ml). Representative data of two experiments are shown. **Figure S4.** The role of platelets in histone-induced death. Mice were injected with either anti-GP-Ib IgG or control IgG two hours before the histone injection (80 µg/g, n = 12 per group). Platelet depletion protected mice from the histone-induced early phase death.**Figure S5.** (A) Hematoxylin and eosin staining of lung sections from mice 10 minutes after injection with vehicle (n = 3), histones (75 µg/g, n = 5), or histones + rTM (110 µg/g, n = 4). rTM prevented histone-induced hemorrhage. Scale bars = 100 µm. (B) Immunoblotting analysis of serum histone H3. Serum samples were collected from mice 10 minutes after injection with histones (75 µg/g) alone and histones + rTM (110 µg/g). Histone H3 degradation products were not detected in mice treated with rTM. S10 indicates 10 µg/ml of recombinant histone H3 diluted in normal mouse serum.
(PDF)

Movie S1 **Echocardiography in mice before histone injection.** This movie consists of three tandem repeats of two heart beats.
(M2V)

Movie S2 **Echocardiography in mice 17 min after histone injection.** Extracellular histones caused dilatation of right ventricle and displacement of interventricular septum toward left ventricle. This movie consists of three tandem repeats of two heart beats.
(M2V)

Movie S3 **Echocardiography in mice 21 min after histone injection.** Extracellular histones caused ventricular arrest. This movie consists of three tandem repeats of one heart beat.
(M2V)
