# Supplementary material for: Recombinant Thrombomodulin Protects Mice against Histone-Induced Lethal Thromboembolism
Source: PLoS One. 2013 Sep 30;8(9):e75961. doi: 10.1371/journal.pone.0075961 (PMC3786915; doi:10.1371/journal.pone.0075961)
Supplement: File S1 — Supporting Figures. Figure S1. (A) The purity of unfractionated calf thymus histones used in this study. Purified unfractionated calf thymus histones (Hs), recombinant histone H3, H4, H2A, and H2B were subjected to SDS-PAGE followed by CBB staining (left panel). M: Molecular weight markers. The identity of each band was confirmed by immunoblotting (right panel). (B) Serum histone H3 levels in mice 10 minutes after histone injection (0-80 µg/g body weight). Serum histone H3 levels were determined by immunoblotting analysis with reference to standard curves of recombinant histone H3 diluted in normal mouse serum at the indicated concentrations. Injection of 20-80 µg/g of histones resulted in serum histone H3 levels of 1-10 µg/ml. Figure S2. Blood smears of mice 10 minutes after histone injection. Histone injection (80 µg/g) eliminated platelets and yielded fragmented red blood cells (arrow heads). Scale bars = 20 µm. Figure S3. Histone-induced platelet aggregation. (A) Washed platelets were stimulated with histone H4 or H3 (10-30 µg/ml). Representative data of three experiments are shown. (B) Platelet-rich plasma anticoagulated with citrate (left panel) or hirudin (right panel) was stimulated with histone H4 (30-100 µg/ml). Representative data of two experiments are shown. Figure S4. The role of platelets in histone-induced death. Mice were injected with either anti-GP-Ib IgG or control IgG two hours before the histone injection (80 µg/g, n = 12 per group). Platelet depletion protected mice from the histone-induced early phase death.Figure S5. (A) Hematoxylin and eosin staining of lung sections from mice 10 minutes after injection with vehicle (n = 3), histones (75 µg/g, n = 5), or histones + rTM (110 µg/g, n = 4). rTM prevented histone-induced hemorrhage. Scale bars = 100 µm. (B) Immunoblotting analysis of serum histone H3. Serum samples were collected from mice 10 minutes after injection with histones (75 µg/g) alone and histones + rTM (110 µg/g). Histone H3 degrada [file pone.0075961.s001.pdf]

Figure S1

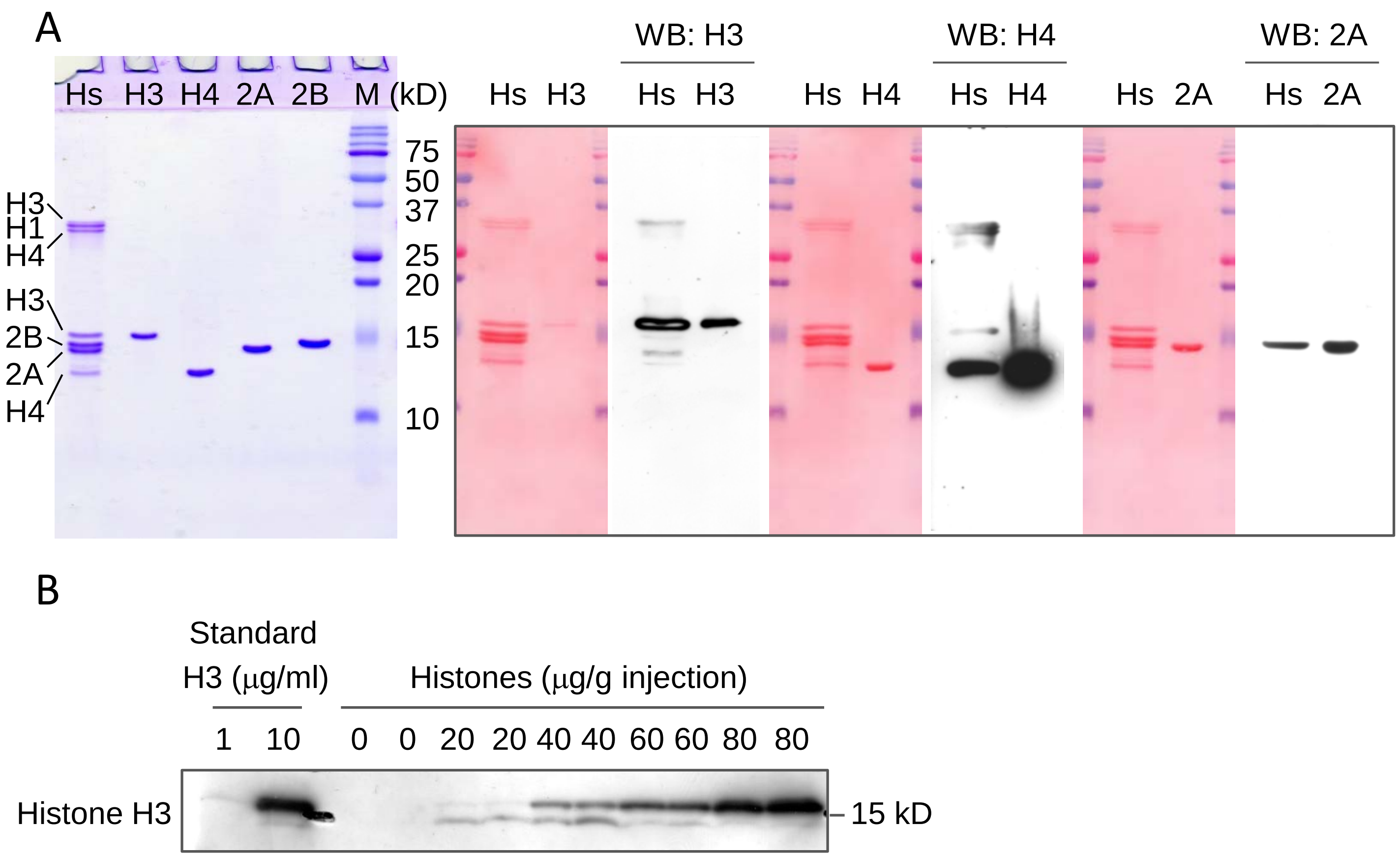

Figure S1. (A) The purity of unfractionated calf thymus histones used in this study. Purified unfractionated calf thymus histones (Hs), recombinant histone H3, H4, H2A, and H2B were subjected to SDS-PAGE followed by CBB staining (left panel). M: molecular weight markers. The identity of each band was confirmed by immunoblotting (right panel). (B) Serum histone H3 levels in mice 10 minutes after histone injection (0-80 μg/g body weight). Serum histone H3 levels were determined by immunoblotting analysis with reference to standard curves of recombinant histone H3 diluted in normal mouse serum at the indicated concentrations. Injection of 20-80 μg/g of histones resulted in serum histone H3 levels of 1-10 μg/ml.

Figure S2

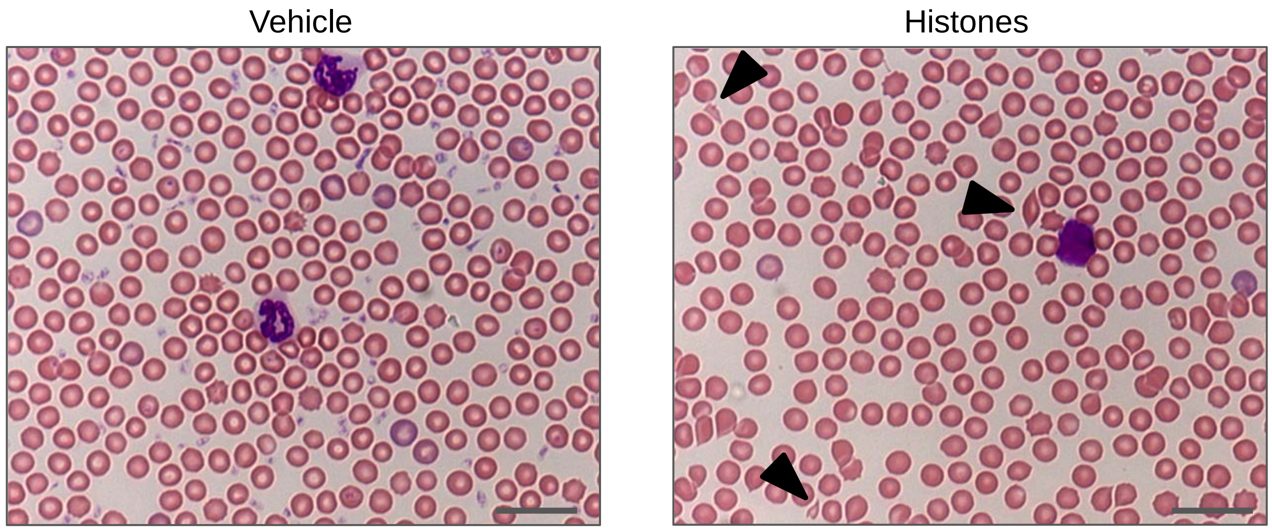

Figure S2. Blood smears of mice 10 minutes after histone injection. Histone injection (80 μg/g) eliminated platelets and yielded fragmented red blood cells (arrow heads). Scale bars = 20 μm.

Figure S3

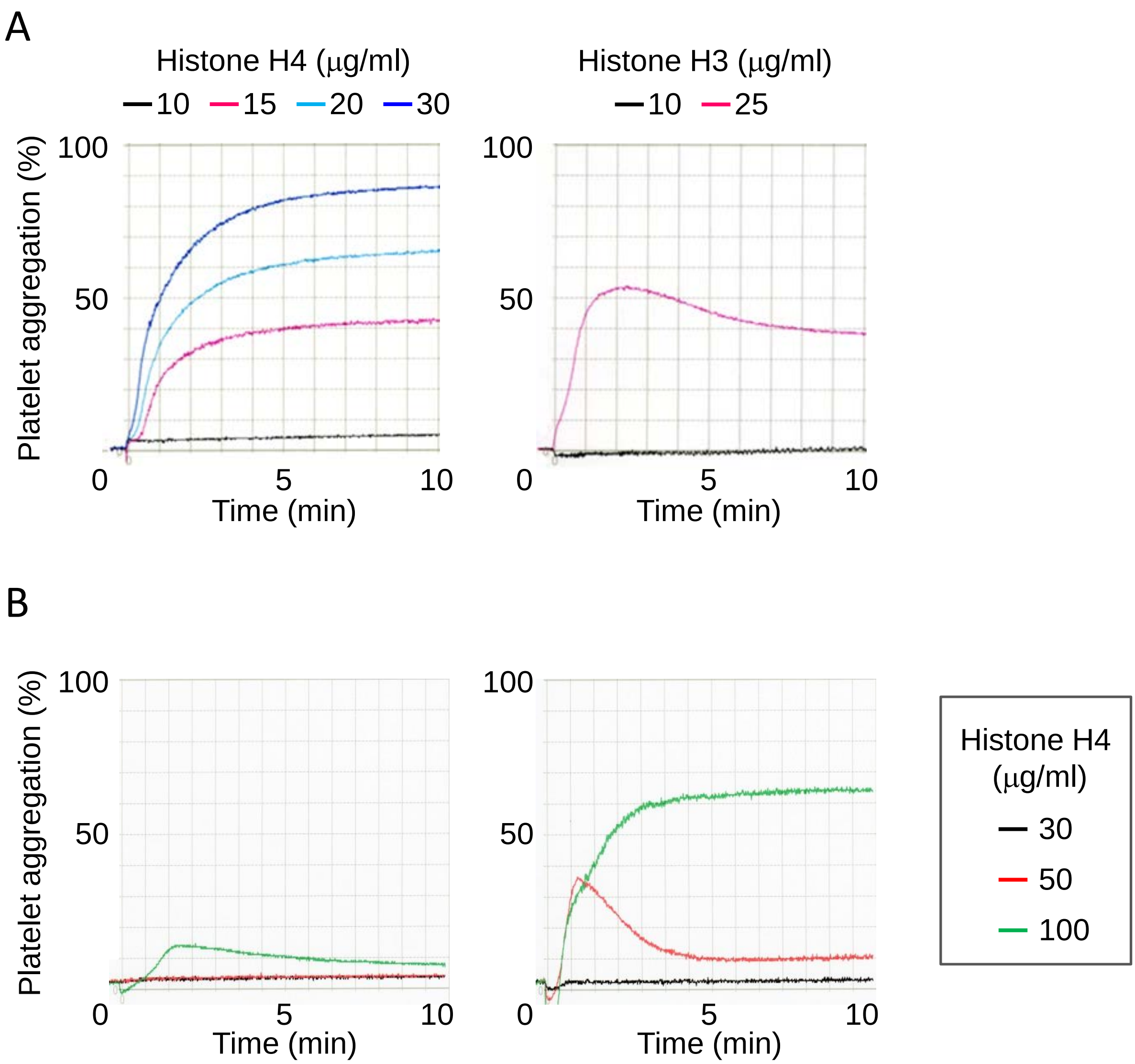

Figure S3. Histone-induced platelet aggregation. (A) Washed platelets were stimulated with histone H4 or H3 (10-30 μg/ml). Representative data of three experiments are shown. (B) Platelet-rich plasma anticoagulated with citrate (left panel) or hirudin (right panel) was stimulated with histone H4 (30-100 μg/ml). Representative data of two experiments are shown.

Figure S4

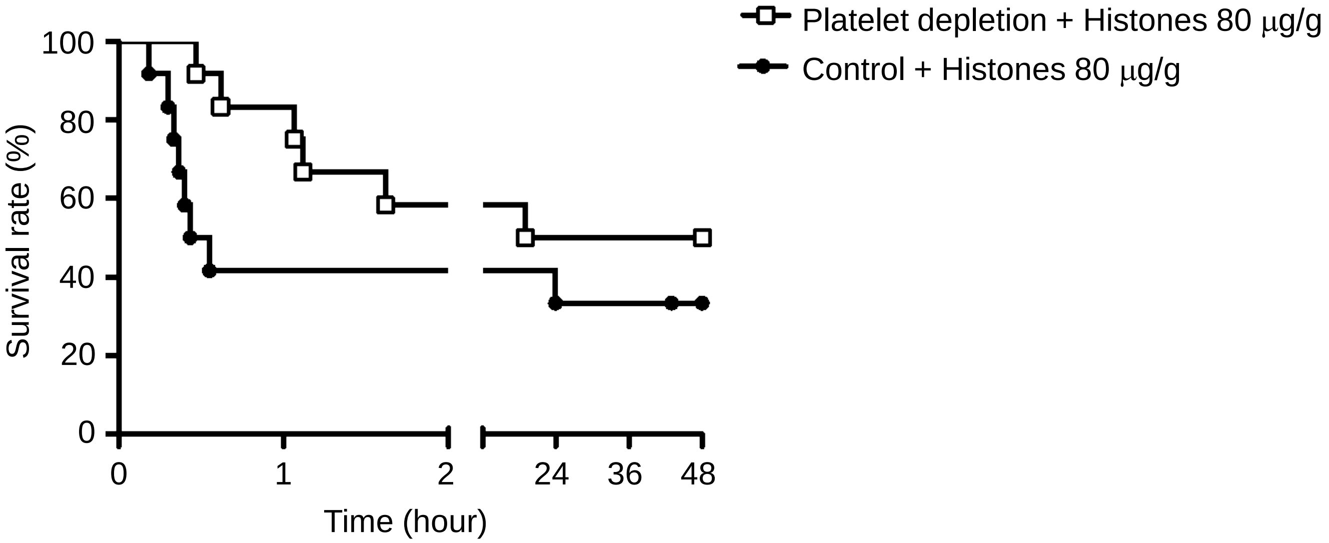

Figure S4. The role of platelets in histone-induced death. Mice were injected with either anti-GP-Ib IgG or control IgG two hours before the histone injection (80 μg/g, n = 12 per group). Platelet depletion protected mice from the histone-induced early phase death.

Figure S5

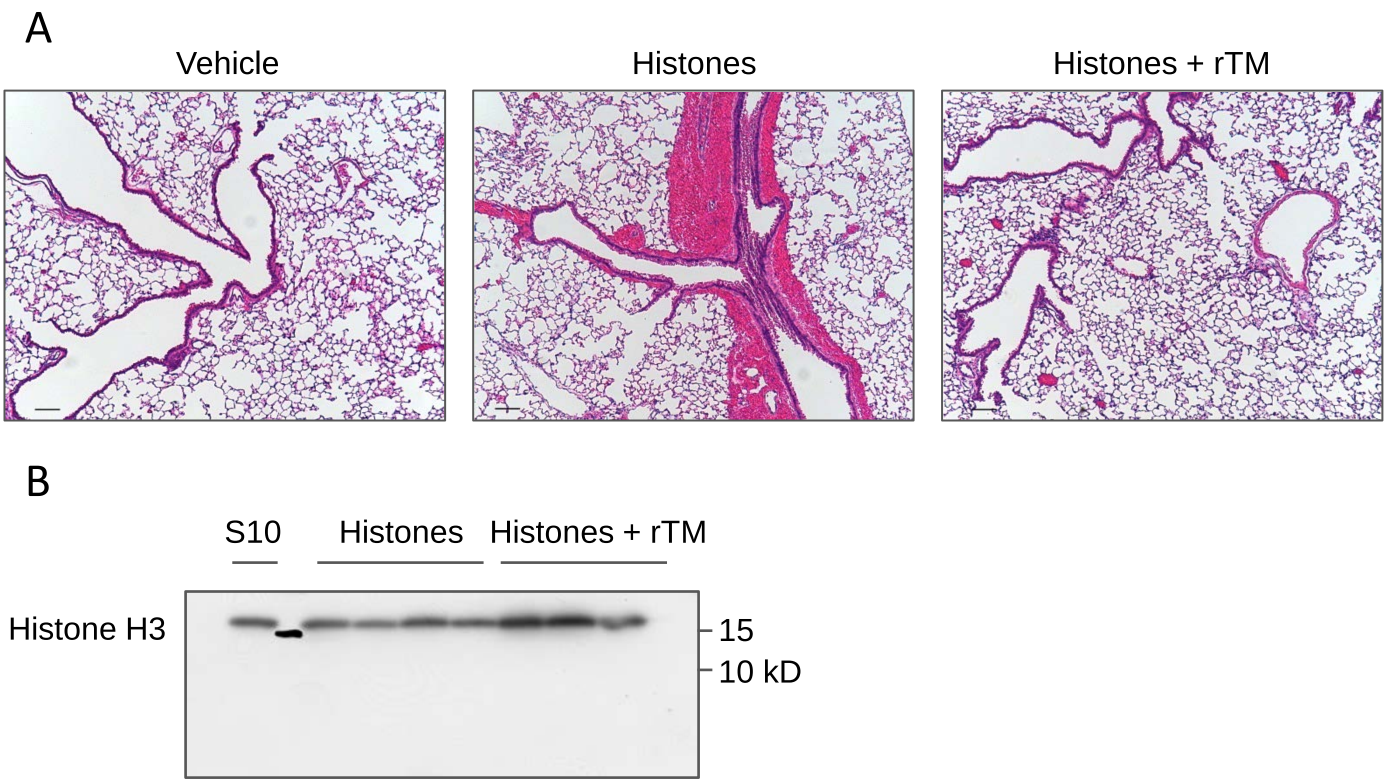

Figure S5. (A) Hematoxylin and eosin staining of lung sections from mice 10 minutes after injection with vehicle (n = 3), histones (75 μg/g, n = 5), or histones + rTM (110 μg/g, n = 4). rTM prevented histone-induced hemorrhage. Scale bars = 100 μm. (B) Immunoblotting analysis of serum histone H3. Serum samples were collected from mice 10 minutes after injection with histones (75 μg/g) alone and histones + rTM (110 μg/g). Histone H3 degradation products were not detected in mice treated with rTM. S10 indicates 10 μg/ml of recombinant histone H3 diluted in normal mouse serum.

Supplementary Movies

Movie S1. Echocardiography in mice before histone injection.

Movie S2. Echocardiography in mice 17 min after histone injection.

Movie S3. Echocardiography in mice 21 min after histone injection.

Extracellular histones caused dilatation of right ventricle and displacement of interventricular septum toward left ventricle. Movies consist of three tandem repeats of one (movie S3) or two (movie S1 and S2) heart beats.
